# Withdrawal from Cocaine Self-administration and Yoked Cocaine Delivery Dysregulates Glutamatergic mGlu_5_ and NMDA Receptors in the Rat Brain

**DOI:** 10.1007/s12640-014-9502-z

**Published:** 2014-11-19

**Authors:** Lucyna Pomierny-Chamiolo, Joanna Miszkiel, Małgorzata Frankowska, Bartosz Pomierny, Ewa Niedzielska, Irena Smaga, Fabio Fumagalli, Małgorzata Filip

**Affiliations:** 1 Department of Toxicology, Department of Biochemical Toxicology, Faculty of Pharmacy, Jagiellonian University, Medical College, Medyczna 9, 30-688 Kraków, Poland; 2 Laboratory of Drug Addiction Pharmacology, Department of Pharmacology, Institute of Pharmacology Polish Academy of Sciences, Smętna 12, 31-343 Kraków, Poland; 3 Dipartimento di Scienze Farmacologiche e Biomolecolari, Università degli Studi di Milano, Via Balzaretti 9, 20133 Milan, Italy; 4 Collaborative Center of Department of Antidrug Policies, Presidency of the Council of Ministers, Roma, Italy

**Keywords:** Cocaine self-administration, Metabotropic glutamate receptors, Ionotropic glutamate receptors, Homer1b/1c, Extinction

## Abstract

In human addicts and in animal models, chronic cocaine use leads to numerous alterations in glutamatergic transmission, including its receptors. The present study focused on metabotropic glutamatergic receptors type 5 (mGluR_5_) and N-methyl-D-aspartate receptor subunits (NMDAR: GluN1, GluN2A, GluN2B) proteins during cocaine self-administration and after 10-day of extinction training in rats. To discriminate the contingent from the non-contingent cocaine delivery, we employed the “yoked”-triad control procedure. Protein expression in rat prefrontal cortex, nucleus accumbens, hippocampus, and dorsal striatum was determined. We also examined the Homer1b/c protein, a member of the postsynaptic density protein family that links NMDAR to mGluR_5_. Our results revealed that cocaine self-administration selectively increased GluN1 and GluN2A subunit in the rat hippocampus and dorsal striatum, respectively, while mGluR_5_ protein expression was similarly increased in the dorsal striatum of both experimental groups. Withdrawal from both contingent and non-contingent cocaine delivery induced parallel increases in prefrontal cortical GluN2A protein expression, hippocampal mGluR_5_, and GluN1 protein expression as well as in accumbal GluN1 subunit expression, while the mGluR_5_ expression was reduced in the prefrontal cortex. Extinction training in animals with a history of cocaine self-administration resulted in an elevation of the hippocampal GluN2A/GluN2B subunits and accumbal mGluR_5_, and in a 50 % decrease of mGluR_5_ protein expression in the dorsal striatum. The latter reduction was associated with Homer1b/1c protein level decrease. Our results showed that both contingent and non-contingent cocaine administration produces numerous, brain region specific, alterations in the mGluR_5_, NMDA, and Homer1b/1c protein expression which are dependent on the modality of cocaine administration.

## Introduction

Cocaine belongs to the most commonly used illicit stimulant drug in Europe with about 2.2 million young drug adults aged 15–34 in 2012 (EMCDDA, 2014). At the global level, the number of adult cocaine users (age 15–64) ranges from 13.3 to 19.7 million (UNDCP, 2012): this renders cocaine use disorder a significant health problem resulting in a large number of medical, psychological, and social problems (Kim and Lawrence 2014).

Cocaine addiction is associated with enduring neurochemical differences in the brain, including glutamate (Glu) neurotransmission (for review see: Pomierny-Chamiolo et al. 2014). In fact, in preclinical models, the expression of drug seeking, modeling the core feature of cocaine addiction, promotes Glu release in the ventral tegmental area and the core of the nucleus accumbens (Madhavan et al. 2013). The release of Glu during cocaine seeking also elicits rapid postsynaptic changes in proteins regulating Glu signaling and surface spine morphology, while attenuation of Glu transmission reduces drug reinforcement and relapse-like behavior (McFarland et al. 2003; Brebner et al. 2005; Gipson et al. 2013). The potentiation of Glu transmission, from prefrontal glutamatergic neurons to the accumbal core during drug-seeking behaviors, is also critical to drug-associated memories (Kalivas 2009). On the other hand, withdrawal from cocaine treatment alters the number of accumbal Glu synapses and spine density associated with a deteriorating actin cytoskeleton and a reduction in Glu signaling-related proteins (Shen et al. 2009). As shown in rodents, these changes are the consequence of reduced basal accumbal extracellular Glu, together with changes in mechanisms responsible for Glu clearance (Pomierny-Chamiolo et al. 2014).

Cocaine-induced fluctuations in glutamatergic transmission contribute to significant adaptations in glutamatergic receptors, including ionotropic N-methyl-D-aspartate receptors (NMDAR) and metabotropic glutamatergic receptors type 5 (mGluR_5_) in cortical and subcortical brain areas (Volkow et al. 2003; Tzschentke and Schmidt 2003) NMDARs are ionotropic channels formed by the combination of different subunits. The obligatory GluN1 is a channel-forming subunit and a site which binds co-agonists glycine or d-serine, GluN2A-D subunits have an agonist site for glutamate and other receptor agonists and antagonists, while GluN3A and B subunits may reduce Ca^2+^ permeability and Mg^2+^ sensitivity (Low and Wee 2010).

Both mGluR_5_ and NMDA receptors seem to be the key players in drug addiction. At the behavioral level, mGluR_5_ knockout mice do not acquire cocaine self-administration response, while blockade of the mGluR_5_ reduces cocaine self-administration in rats, cocaine-induced lethality as well as expression of behavioral sensitization in mice (Keck et al. 2014; Pomierny-Chamiolo et al. 2014). Cornish et al. (1999) reported that stimulation of NMDARs in the nucleus accumbens augments the reinforcing effect of cocaine, while inhibition of GluN2B by ifenprodil-blocked cocaine sensitization effect (Schumann and Yaka 2009).

**Fig. 1:**
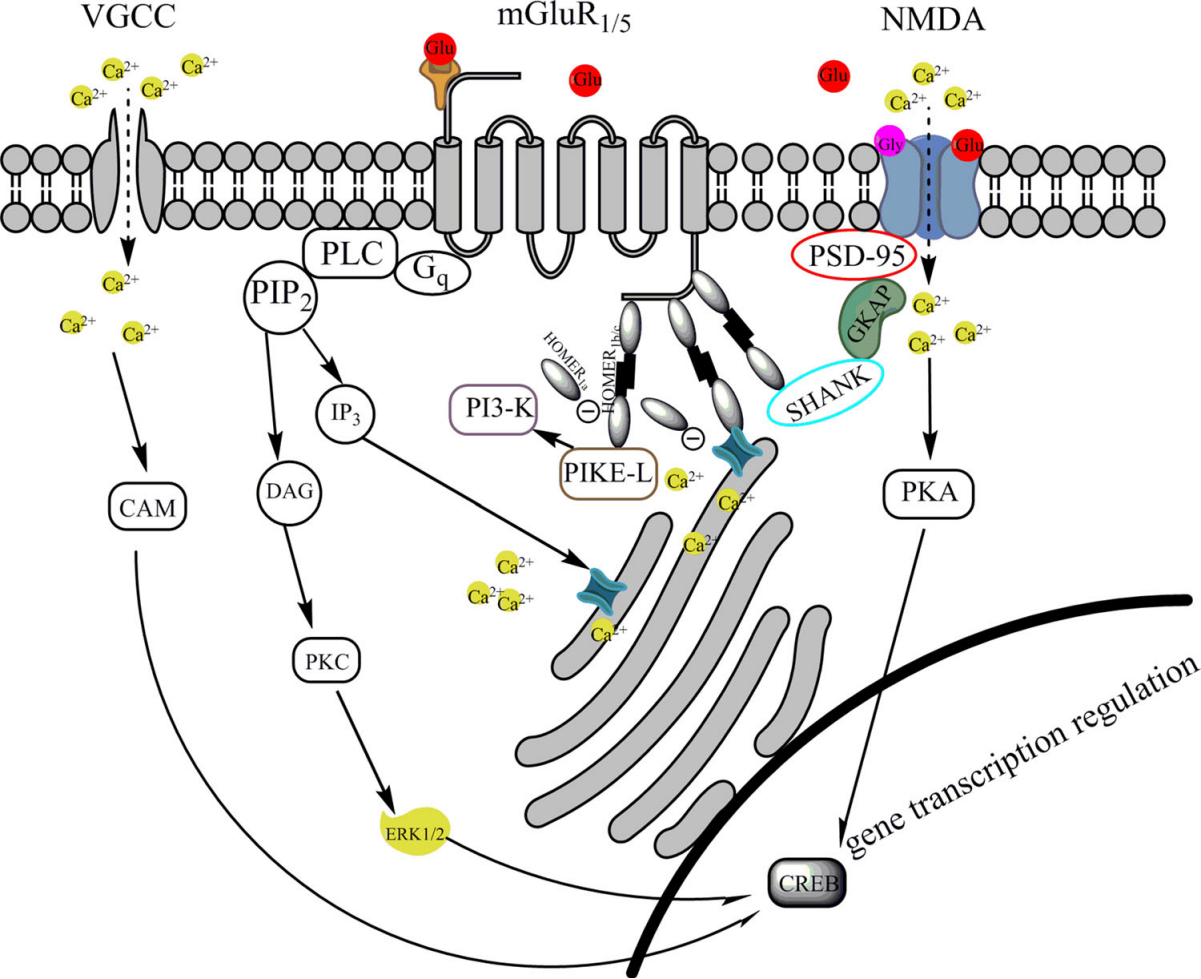
Interactions between mGluR_1/5_ and NMDA receptors and their signaling pathways in the postsynaptic neuron. NMDA receptors are linked to mGluR_1/5_ receptors through complex composed of cytoplasmatic proteins PSD-95, guanylate kinase-associated protein (GKAP), Shank protein and Homer1b/c. Stimulation of mGluR_1/5_ receptors activates phospholipase C (PLC) and thereby leads to enhanced production of inositol-1,4,5-triphosphate (IP3) and diacylglycerol (DAG) forms phosphatidylinositol-4,5-biphosphate (PIP_2_) and potentiates L-type voltage-dependent calcium channels (VGCC). Elevated IP_3_ level mobilizes Ca^2+^ release from internal stores. DAG contributes to activation protein kinase C (PKC) and subsequently extracellular signal-regulated protein kinases ERK1/2 phosphorylation. mGluR_1/5_ receptors are also coupled by means of Homer1b/c and phosphoinositide-3-kinase enhancer (PIKE-L) to phosphoinositide-3-kinase (PI3-K). All interactions involving long isoforms of Homer are disrupted by the short isoform Homer_1a_ and thereby affect mGluR_1/5_ signaling. Summarizing activation of mGluR_5_ and NMDA receptors leads to phosphorylation transcription factors, therein cAMP response element-binding protein (CREB) and changes in gene expression

NMDAR and mGluR_5_ enter into mutual interactions (Fig. 1). Connection between these receptors regulates the plasma membrane trafficking of them via the complex Shank-GKAP-PSD95 connected to the NMDAR (Scheggi et al. 2002; Ferraguti et al. 2008; Szumlinski et al. 2008; Ghasemzadeh et al. 2009a). At the functional level, it was reported that mGluR_5_ desensitization is mediated by activation of NMDAR (Alagarsamy et al. 2005). mGluR_5_-Homer-NMDA complex is importantly involved in synaptic plasticity such as LTP and LTD in drug addiction (Anwyl 1999; Ferraguti and Shigemoto 2006; Ronesi and Huber 2008; Kasanetz et al. 2010; Brown et al. 2011; Huang et al. 2011; Shen and Kalivas 2013).

Based on these lines of evidence, we decided to investigate more in details the expression of these receptors using a different approach. The unique aspect of the current study was the investigation of the effects of contingent and non-contingent cocaine delivery as well as postcocaine drug free period with extinction training sessions (either 1 or 10 days) on the mGluR5-Homer-NMDAR in order to discriminate between the motivational and the pure pharmacological properties of cocaine. To extend few preclinical studies on the NMDAR and mGluR_5_ distribution after cocaine administration (Ghasemzadeh et al. 1999; Crespo et al. 2002; Hemby et al. 2005; Ary and Szumlinski 2007; Ghasemzadeh et al. 2011), we studied several rat brain areas related to reward processing (the nucleus accumbens), habit forming learning (the dorsal striatum), executive control (the prefrontal cortex), as well as learning and memory (the hippocampus) (Kalivas 2004).

## Methods

### Animals

Naive, male Wistar rats (280–310 g; Charles River, Germany) were housed individually in standard plastic rodent cages in a room maintained at 20 ± 1 °C and 40–50 % humidity under a 12-hour light–dark cycle (lights on at 6.00 a.m.) with free access to water and standard animal food during habituation period. Following habituation, rats were maintained on limited access to water during the initial (2 days) lever press training sessions. All procedures were conducted during the light phase of the light–dark cycle (between 8.00 a.m. and 3.00 p.m.). All the experimental procedures were carried out in accordance with the NIH Guide for the Care and Use of Laboratory Animals and with approval of the Animal Care and Use Committee at the Institute of Pharmacology, Polish Academy of Sciences in Krakow.

### Drugs

Cocaine HCl (Toronto Research, Canada) was dissolved in sterile 0.9 % NaCl and given intravenously (0.1 ml/infusion).

### Behavioral Experiments

#### Initial Training Sessions

Rats were trained to press the lever of standard operant conditioning chambers (Med-Associates, USA) under a fixed ratio (FR) 5 schedule of water reinforcement, as described previously (Pomierny-Chamiolo et al. 2013).

#### Surgery

Two days following lever press training and free access to water, the rats were anesthetized with ketamine HCl (75 mg/kg, Bioketan; Biowet, Puławy, Poland) and xylazine (5 mg/kg, Sedazin; Biowet, Puławy, Poland) and chronically implanted with catheters in the external jugular vein. The catheters were flushed every day with 0.1 ml of saline solution containing heparin (70 U/ml, Biochemie GmbH, Kundl, Austria) and 0.1 ml of solution of cefazolin (10 mg/ml; Biochemie BmbH, Austria). Catheter potency was tested periodically, or whenever an animal displayed behavioral outside baseline parameters.

#### Cocaine Self-administration Procedure

**Fig. 2:**
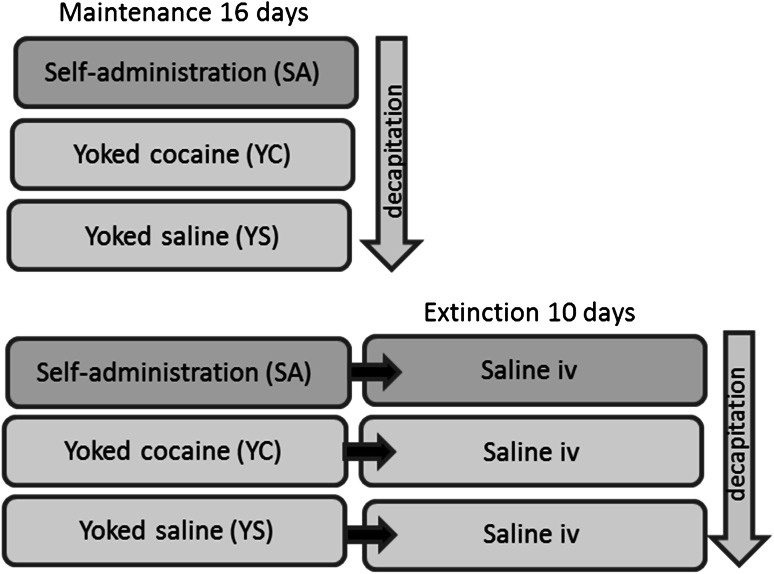
Experimental design

The procedure was carried out as described previously (Bystrowska et al. 2014). Cocaine (0.5 mg/kg/infusion) self-administration was conducted under FR5 schedule of reinstatement for 16-daily 2-h sessions. Animals were divided into two subgroups (see experimental design in Fig. 2). One of them (*n* = 8–10 rats/group) was sacrificed immediately after last cocaine self-administration session, while other group (*n* = 8–10 rats/group) was underwent extinction training.

#### Extinction Procedure

During extinction, saline was delivered and there was no presentation of the conditioned stimulus during 2-h daily sessions. On the 10th day of extinction, animals that met the extinction criterion (i.e., responses on the active lever fell to <10 % of the responses at the active lever reached during maintenance) were sacrificed immediately following the session.

#### “Yoked” Procedure

In the experiment, we used the “yoked” procedure, to distinguish between the pharmacological and motivational effects of cocaine intake. In this procedure, each rat actively self-administering cocaine had assigned other two rats passively receiving either cocaine or saline.

#### Dissection

**Fig. 3:**
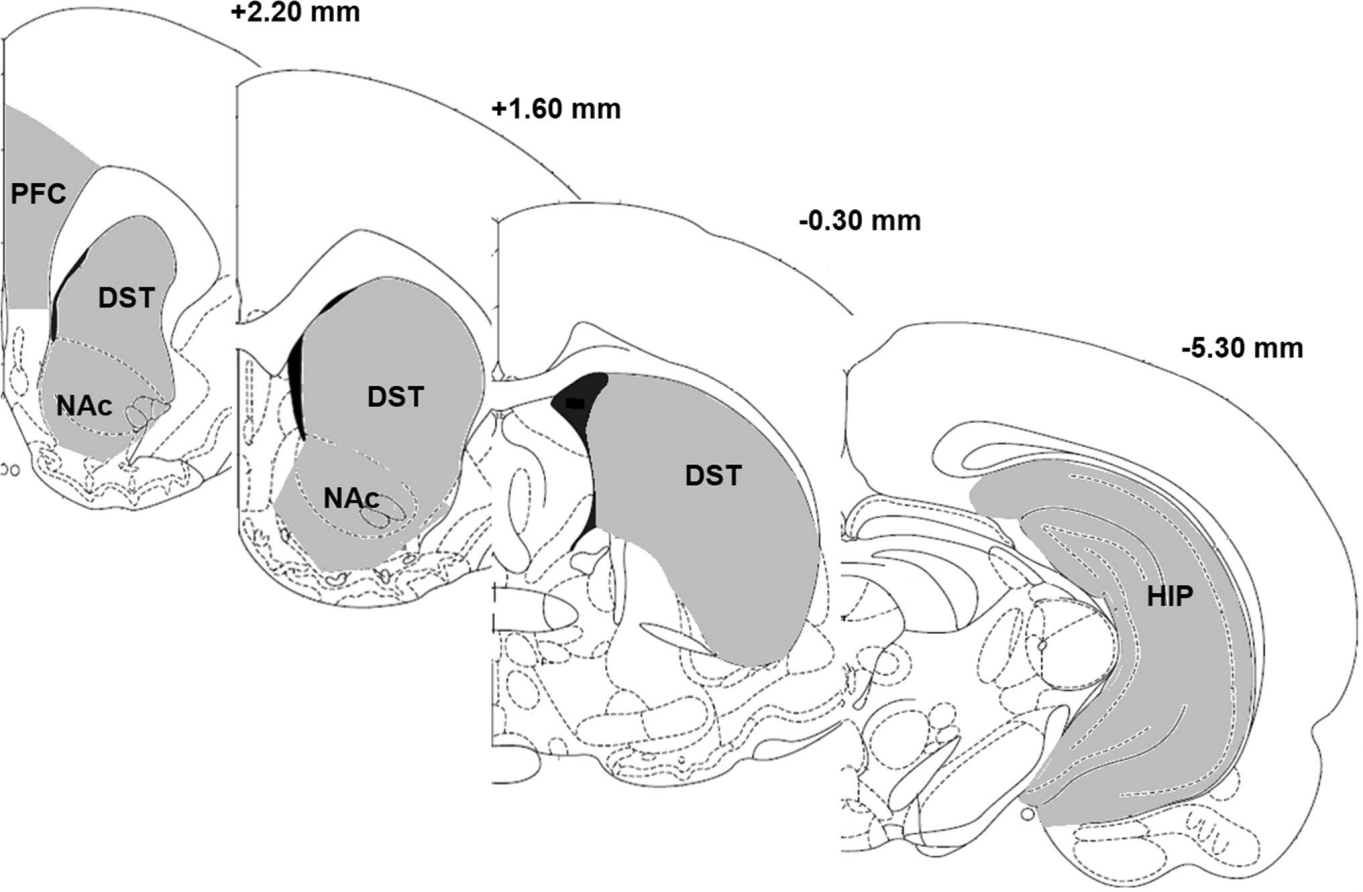
The dissection of the prefrontal cortex, hippocampus, nucleus accumbens, and dorsal striatum. The numbers on the coronal brain section represent distance from Bregma

After decapitation, the brain was quickly removed and chilled in ice-cold saline. The prefrontal cortex, hippocampus, dorsal striatum, and nucleus accumbens from each animal were dissected out (Paxinos and Watson 1998) (Fig. 3). Samples were immediately frozen on dry ice, and stored at −80 °C.

### Western Blot Analysis

Dissected brain structures were homogenized in 2 % sodium dodecyl sulfate (SDS) with protease and phosphatase inhibitor cocktails, 20 mM NaF, 1 mM phenylmethylsulfonyl fluoride (PMSF), and 1 mM Na_3_VO_4_ (Sigma Aldrich, USA) using Ultra-Turrax homogenizer (10 s at 10,000 rpm), sonicated and then denatured for 10 min at 95 °C. After, insoluble material was removed by centrifugation at 10,000 rpm under 4 °C for 10 min. For protein determination, a bicinchoninic acid assay (BCA) protein assay kit (Thermo Scientific, Rockford, IL, USA) was used. Protein samples (35 µg) were resolved by 8 % SDS polyacrylamide gels and transferred to a polyvinylidene difluoride (PVDF) membrane. Membranes were blocked in 5 % non-fat dry milk, and separate sets of membranes were probed with rabbit anti-mGluR_5_ polyclonal antibody (1:2000) or mouse anti-Homer1b/c monoclonal antibody (1:500). Blots were washed and incubated in horseradish peroxidase (HRP)-conjugated secondary antibody (1:7500). Bands were detected with the ECL method using Western Bright Quantum chemiluminescent substrate (Advansta Inc., USA) and were imaged in G:Box (Syngene, USA). Next membranes were stripped using Restore Western Blot Stripping Buffer (Thermo Scientific, USA), blocked in 5 % non-fat dry milk in Tris-Buffered Saline and Tween 20 mixture (TBST) and re-probed with mouse monoclonal anti-β-actin (1:1000) or goat polyclonal anti-NR1 (1:500) using the same protocol as before. After washes, the blots were incubated in goat anti-mouse secondary antibody (1:5000) or donkey anti-goat (1:5000). After visualization of these proteins, sets of membranes were used to probe for GluN2A (mouse monoclonal; 1:500) and after then GluN2B (rabbit polyclonal; 1:400). The data were analyzed with Gene Tools (version 4.03 (a); Syngene, USA). The expressions of mGluR_5_, GluN1, GluN2A, GluN2B, or Homer1b/c were evaluated relative to that of ß-actin. All antibodies were obtained from Santa Cruz Biotechnology (USA), except anti-mGluR_5_ delivered from Millipore Bioscience Research Reagents (USA).

### Data Analysis

In the behavioral procedures, the data were analyzed by Student *t* test (number of active and passive lever presses, infusions). In the biochemical assays, one-way ANOVA, followed by posthoc Newman–Keuls’ test, was applied to evaluate statistically significant differences between the treatment groups. To separate the effects of treatment (saline vs. cocaine) and the way of drug intake (self vs. yoked), we also used the Student *t* test. The criterion for statistically significant differences was set at *p* < 0.05.

## Results

### Behavioral Studies

**Fig. 4:**
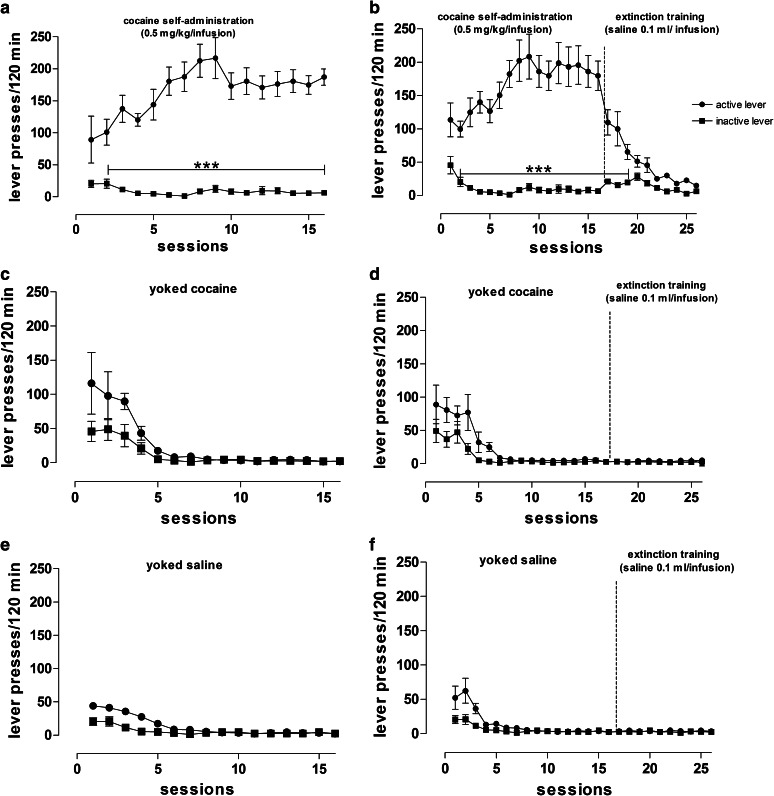
The number of active and inactive lever presses in rats that acquired and maintained cocaine (0.5 mg/kg/infusion) self-administration (*left panels*: **a**, **c**, **e**) and animals that underwent 10-day extinction training (*right panels*: **b**, **d**, **f**) with their yoked controls that received passive infusions of cocaine or saline. Data are presented as the mean ± SEM from 8 rats/group, ***p < 0.0001 versus inactive lever

After 16 sessions of self-administration, the rats showed stable responding on levers during the last 6 self-administration maintenance sessions with less than a 10 % difference in their daily intake of cocaine. Rats responded significantly more frequently on the active lever than on the inactive lever from the 2nd to 16th experimental session (*p* < 0.0001) (Fig. 4a, b). The rats self-administered either 24–37 injections of cocaine (daily mean cocaine intake between 12 and 18.5 mg/kg; group 1) or 21–39 injections (daily mean cocaine intake between 10.5–19.5 mg/kg; group 2). During 16 experimental cocaine self-administered sessions, animals received approximately 272 or 281 mg/kg/rat.

During the extinction phase, neither the drug nor the drug-paired stimuli was given in response to lever pressing, which resulted in decrease in active lever presses (Fig. 4b). From day 19 to day 26 of experiment, the difference between responding on the active versus the inactive lever failed to reach significance (Fig. 4b).

In the yoked cocaine group (Fig. 4c, d), the total number of active lever presses did not differ from inactive lever. These animals passively received exactly the same amount of cocaine at the same time as the rats that learned to actively self-inject cocaine.

In the yoked saline group (Fig. 4e, f), the difference between responding on the active versus the inactive lever failed to reach significance.

### Biochemical Studies

**Fig. 5:**
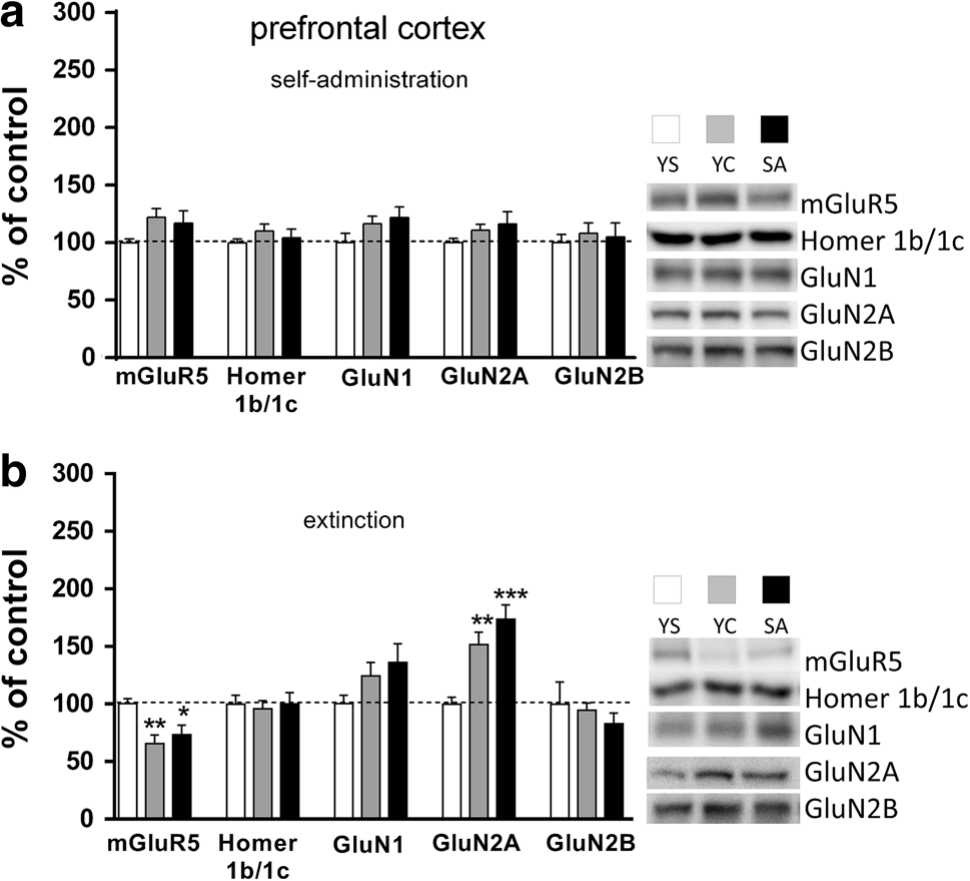
Effects of cocaine self-administration (**a**) and extinction training (**b**) on the mGluR_5_, Homer1b/1c, and NMDA (subunits: NR1, NR2A, NR2B) receptor protein expression in the rat prefrontal cortex with a “yoked” control procedure. Data were normalized to saline-treated animals (% of yoked saline; YS) and represent the mean (±SEM) of 8–10 animals/group. *p < 0.05, **p < 0.01, ***p < 0.001 versus YS. On the *right*, representative immunoblots for the protein levels of mGluR_5_, Homer1b/1c, NR1, NR2A, and NR2B are shown

**Fig. 6:**
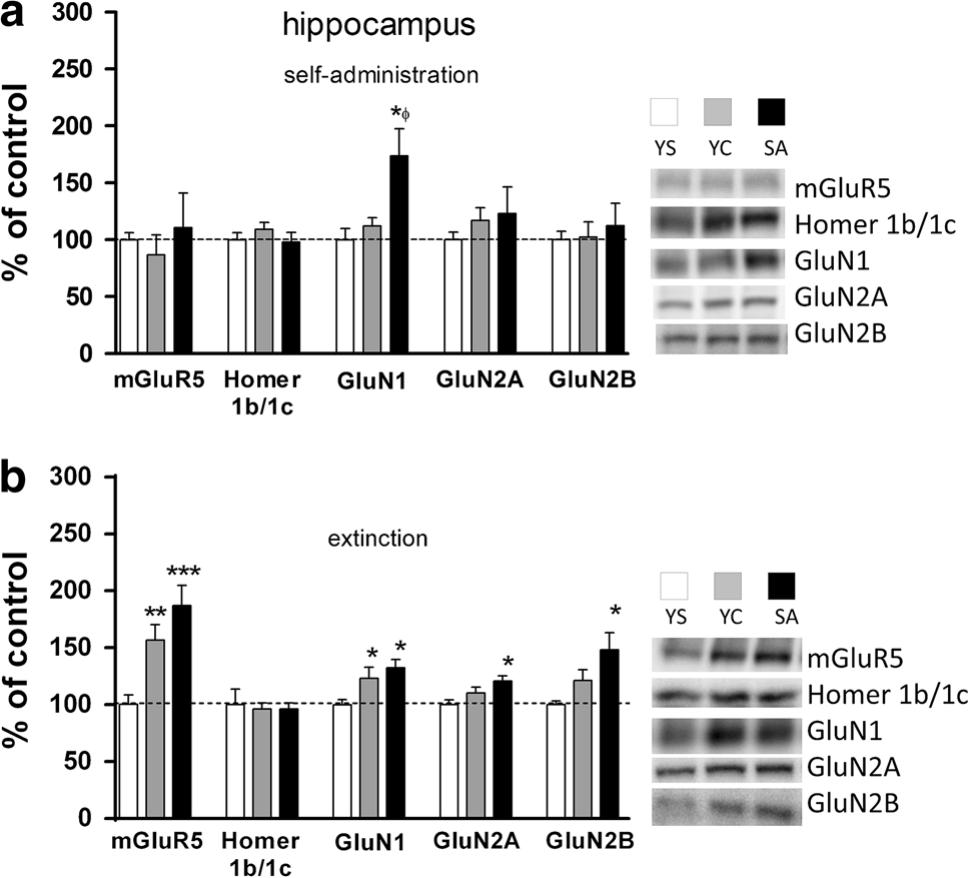
Effects of cocaine self-administration (**a**) and extinction training (**b**) on the mGluR_5_, Homer1b/1c, and NMDA (subunits: NR1, NR2A, NR2B) receptor protein expression in the rat hippocampus with a “yoked” control procedure. Data were normalized to saline-treated animals (% of yoked saline; YS) and represent the mean (±SEM) of 8–10 animals/group. *p < 0.05, **p < 0.01, ***p < 0.001 versus YS. ^ϕ^p < 0.05 versus YC. On the *right*, representative immunoblots for the protein levels of mGluR_5_, Homer1b/1c, NR1, NR2A, and NR2B are shown

**Fig. 7:**
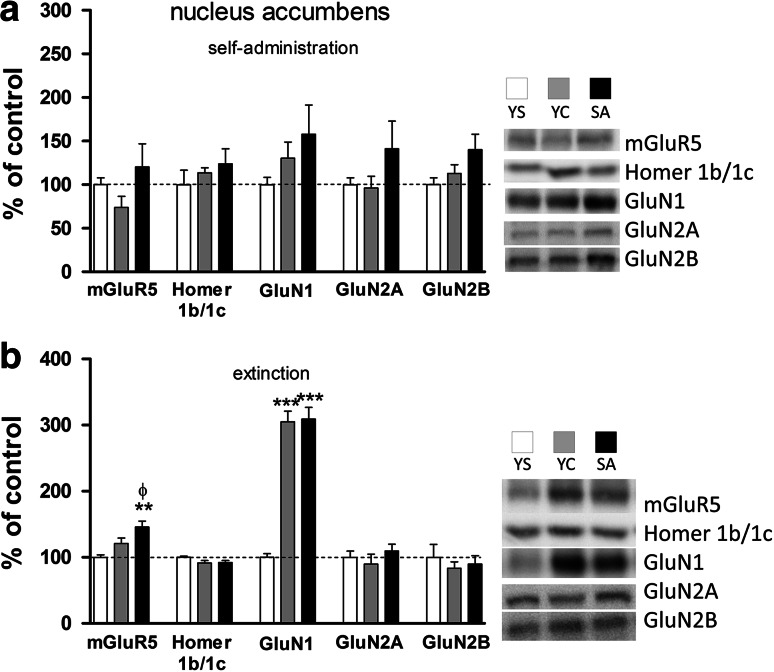
Effects of cocaine self-administration (**a**) and extinction training (**b**) on the mGluR_5_, Homer1b/1c, and NMDA (*subunits*: NR1, NR2A, NR2B) receptor protein expression in the rat nucleus accumbens with a “yoked” control procedure. Data were normalized to saline-treated animals (% of yoked saline; YS) and represent the mean (±SEM) of 8–10 animals/group. **p < 0.01, ***p < 0.001 versus YS; ^ϕ^p < 0.05 versus YC. On the *right*, representative immunoblots for the protein levels of mGluR_5_, Homer1b/1c, NR1, NR2A, and NR2B are shown

**Fig. 8:**
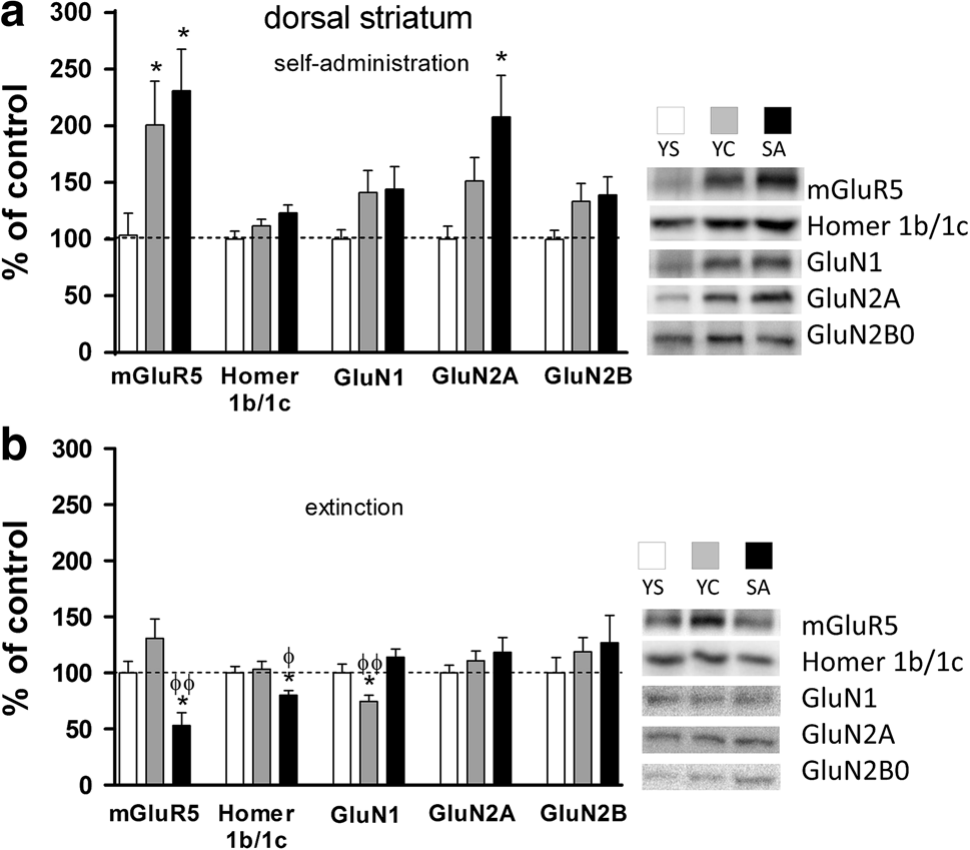
Effects of cocaine self-administration (**a**) and extinction training (**b**) on the mGluR_5_, Homer1b/1c, and NMDA (*subunits*: NR1, NR2A, NR2B) receptor protein expression in the rat dorsal striatum with a “yoked” control procedure. Data were normalized to saline-treated animals (% of yoked saline; YS) and represent the mean (±SEM) of 8–10 animals/group. *p < 0.05 versus YS; ^ϕ^p < 0.05, ^ϕϕ^p < 0.01 versus YC. On the *right*, representative immunoblots for the protein levels of mGluR_5_, Homer1b/1c, NR1, NR2A, and NR2B are shown

The data of protein expression for mGluR_5_, Homer1b/c, and NMDA receptor subunits in rat brain structures in maintenance phase and after extinction training are presented in Figs. 5, 6, 7, 8.

### mGluR_5_ Expression

In rats trained to self-administer cocaine (0.5 mg/kg/infusion) and those given yoked cocaine injections, a one-way ANOVA analysis showed a significant effect for mGluR_5_ protein expression (F(2,23) = 4.811, *p* = 0.0314) in the dorsal striatum. The posthoc test revealed that mGluR_5_ protein expression was significantly higher in both cocaine groups compared to yoked saline group (*p* < 0.05). (Figure 8a). A 20 % non-significant trend to increase mGluR_5_ protein level was also observed in the prefrontal cortex (Fig. 5a).

In animals that underwent forced abstinence from cocaine active and yoked administration, there was a significant effect in mGluR_5_ protein expression in the prefrontal cortex as shown by a one-way ANOVA (F(2,23) = 6.973, *p* = 0.048). Posthoc analyses revealed a significant decrease (ca. 30–35 %) in rats previously self-administered cocaine (*p* < 0.05) and those treated passively with the drug (*p* < 0.01) in comparison to yoked saline group (Fig. 5b). In the same animals, an opposite effect was noted in the hippocampus, as evidenced by a one-way ANOVA analysis (F(2,23) = 10.05, *p* = 0.0009) and by Newman–Keuls’ posthoc data (+55–85 %) in active cocaine group (*p* < 0.001) and yoked cocaine group (*p* < 0.01) versus yoked saline group (Fig. 6b).

A one-way ANOVA showed significant changes during forced cocaine abstinence in mGluR_5_ protein expression in the nucleus accumbens (F(2,23) = 9.637, *p* = 0.0012) and dorsal striatum (F(2,23) = 7.962, *p* = 0.0036). The posthoc analyses revealed a 45 % rise in mGluR_5_ protein expression in the nucleus accumbens (*p* < 0.01 in comparison with yoked saline group, and *p* < 0.05 in comparison with yoked cocaine group) (Fig. 7b). On the other hand, in the dorsal striatum of animals extinguished form cocaine self-administration, a 47 % decrease in mGluR_5_ protein expression (*p* < 0.05) in comparison with saline group and *p* < 0.01 with yoked cocaine group were detected (Fig. 8b).

### NMDA Subunit Expression

A one-way ANOVA analysis showed that cocaine self-administration resulted in changes in GluN1 protein expression in the hippocampus (F(2,23) = 6.484, *p* = 0.0064) (Fig. 6a) and in GluN2A protein level in the dorsal striatum (F(2,23) = 4.591, *p* = 0.0222) (Fig. 8a). The posthoc analyses revealed in rats self-administered cocaine a significant increase in GluN1 subunit protein expression (p < 0.05) in the hippocampus compared to yoked saline and yoked cocaine groups and in the dorsal striatum in GluN2A subunit protein expression (p < 0.05) compared to yoked saline group. Moreover, increasing trends were seen for GluN1, GluN2A-2B subunits in the nucleus accumbens, and in the dorsal striatum for GluN2B subunits (Figs. 7a, 8a).

In animals that extinguished from cocaine active and yoked administration, we found significant changes in NMDA receptor subunits, with more intensive alterations being observed for rats that self-administered cocaine. In those animals, GluN1 subunit expression was elevated in the hippocampus (ANOVA: F(2,23) = 4.966, *p* = 0.0171; posthoc test: *p* < 0.05 versus yoked saline group) (Fig. 6b) and the nucleus accumbens (ANOVA: F(2,23) = 71.42, *p* < 0.0001; posthoc test *p* < 0.001 versus saline group) (Fig. 7b), while in the prefrontal cortex some trends were observed. In the dorsal striatum, we found a significant decrease of GluN1 subunit protein expression in yoked cocaine group (ANOVA: F(2,23) = 8.245, *p* = 0.0023; posthoc test *p* < 0.05 in comparison with yoked saline group and *p* < 0.01 in comparison with yoked cocaine group) (Fig. 8b).

In animals that extinguished from cocaine self-administration, we report a significant rise (ca. 20 %) of GluN2A subunit expression in the hippocampus (ANOVA: F(2,23) = 5.098, *p* = 0.0157; posthoc test: *p* < 0.05) (Fig. 6b), while all rats treated with cocaine showed an enhancement (>50 %) in the prefrontal GluN2A subunit expression (ANOVA: F(2,23) = 14.14, *p* = 0.0001; posthoc test: at least *p* < 0.01 in comparison with yoked saline group) (Fig. 5b).

For GluN2B subunit protein expression, we found a significant (ca. 45 %, *p* < 0.05) rise in the hippocampus of animals withdrawn from cocaine self-administration and a non-significant increase (ca. 27 %) in yoked cocaine controls (ANOVA: F(2,23) = 4.584, *p* = 0.0223) (Fig. 6b).

### Homer1b/1c Expression

As shown in Fig. 8b, only extinction training in rats previously self-administered cocaine produced a 50 % drop in Homer1b/1c protein expression in the dorsal striatum (ANOVA: F(2,23) = 4.514, *p* = 0.0234; posthoc test: *p* < 0.05 versus yoked saline and yoked cocaine groups) (Fig. 8b).

## Discussion

Our results show alterations in mGluR_5_ and NMDAR Protein expression following cocaine and its withdrawal in the rat brain that depends on the type of drug delivery and its withdrawal as well as on brain region.

### Effects of Contingent Cocaine Delivery on mGluR_5_ and NMDAR Protein Expression

Cocaine self-administration increased GluN1 and GluN2A subunit expression in the hippocampus and dorsal striatum, respectively. Marked rises were found also for mGluR_5_ receptor expression in striatum in a way that was independent from the modality of cocaine exposure.

Some previous reports indicate that long-term cocaine self-administration in rats produced an up-regulation of GluN1 gene expression in some limbic and subcortical areas (Crespo et al. 2002), while significant increases in the accumbal GluN1 subunit and a trend in mGluR_5_ were noticed in cocaine overdose victims as well as in rhesus monkeys that self-administered cocaine for 18 months (Hemby et al. 2005). As increases in the hippocampal, dorsal striatal (present study), and accumbal (Fitzgerald et al. 1996) GluN1 and GluN2A subunits have not been seen after passive repeated cocaine administration, the findings may suggest them as molecular mediators of motivational or/and conditioned aspects of drug abuse. In fact, elevated GluN1 and GluN2 subunit levels may represent an initial step in synaptic plasticity associated with cocaine self-administration. From a functional perspective, both subunits are required for the long-term synaptic changes as GluN1 subunit controls Ca^2+^ permeability (Huang et al. 2011), while GluN2 subunits promote LTP (Jin and Feig 2010). Preclinical data on rodents show that NMDAR in the hippocampus is involved in the formation of aversive memory (Bonini et al. 2011), while those localized in striatal areas contribute to the memorization of a complex motor task in which the GluN2A subunits have a critical role in the slow acquisition phase of motor learning (Lemay-Clermont et al. 2011). Further research is warranted to examine whether changes observed in the present paper indicate any type of plasticity.

As found, cocaine delivery evoked increases in mGluR_5_ protein expression. Since we did not address cell surface or intracellular mGluR_5_ localization (Purgert et al. 2014), we also can only speculate about the functional outcomes of such increases. One possibility is that repeated cocaine via mGluR_5_ might alter protein-synthesis-dependent LTD (by intracellular mGluR_5_) or both LTD and LTP (via cell surface mGluR_5_). Another possibility is that, cocaine pharmacological mechanisms trigger the increase in mGluR_5_ availability that reflects—among others—the attention and/or the vigilance of animals. In fact, the density of these receptors is reduced in the brain of patients with depression, particularly in brain structures involved in regulating wakefulness (Deschwanden et al. 2011).

The observed changes in Glu receptor protein expression are probably caused by several mechanisms. Among others, former activation of synaptic DA-ergic, NA-ergic, and 5-HT-ergic transmissions via cocaine’s inhibition of the monoamine transporters may be considered (Gatley et al. 1999; Brown et al. 2001). As reported by our group (Wydra et al. 2013) and other teams (Pettit and Justice 1989; Lecca et al. 2007; Parsons et al. 1996) cocaine self-administered or delivered by yoked passive injections potently increases in vivo extracellular DA and/or 5-HT in the nucleus accumbens. By using genetic and pharmacological tools it was shown that the function and subunit composition of NMDARs are highly influenced by monoamine neurotransmitters (Boyer et al. 1998; Masuko et al. 2004; Yuen et al. 2005; Singh et al. 2013). Increased hippocampal GluN1 as well as striatal GluN2A and mGluR_5_ subunits may also represent a compensatory mechanism to offset the changes in Glu levels. Small increases at a few isolated time points during the self-administration sessions (Sizemore et al. 2000; see also Wydra et al. 2013) as well as decreases in basal Glu extracellular levels of nucleus accumbens in the same behavioral procedure used in the present study were noticed. The engagement of NMDAR and mGluR_5_ to the rewarding effects of cocaine supports preclinical pharmacological analyses (for review see Pomierny-Chamiolo et al. 2014).

### mGluR_5_ and NMDAR Protein Expression Following Extinction Training

Extinction training evoked significant up-regulation of GluN2A in the prefrontal cortex, GluN1 in the hippocampus and nucleus accumbens, while mGluR_5_ was either up-regulated in the hippocampus or down-regulated in the prefrontal cortex. These changes appeared in parallel in cocaine self-administered or yoked cocaine animals. Interestingly, only rats with previous voluntary access to cocaine showed increased GluN2A and GluN2B subunit expression in the hippocampus and mGluR_5_ in the nucleus accumbens; however, the most eminent change in those rats was the reduction in mGluR_5_ protein level in the striatum. The latter reduction was associated with the Homer1b/1c down-regulation.

Consistent with Ghasemzadeh et al. (2011), a decrease in prefrontal mGluR_5_ protein expression may reflect a molecular correlate of relapse liability and a trigger to reinstate drug-seeking behavior. As confirmed by pharmacological and genetic analyses, the receptor constitutive activity appears to protect against stress, cue, or drug priming effect during cocaine seeking in rodents or squirrel monkeys (for review see: Pomierny-Chamiolo et al. 2014; Martin-Fardon et al. 2009; Lee et al. 2005; Iso et al. 2006; Kumaresan et al. 2009; Novak et al. 2010). Moreover, the receptor pharmacological blockade in the ventromedial prefrontal cortex reduced, while local mGluR_5_ stimulation facilitated, extinction learning in cocaine-withdrawn rats (Ben-Shahar et al. 2013). A recent neuroimaging report also indicated a significant reduction in mGluR_5_ binding in the gray matter of human smokers (Akkus et al. 2013). We also demonstrated a drop in mGluR_5_ protein expression in the prefrontal cortex of yoked cocaine animals which were exposed to cues and—despite the possibility to associate the drug cue to the operant response—they formed a Pavlovian association between the cue presentation and cocaine effects which were messed during animals’ exposure to experimental chambers in the absence of cocaine and its cue. As no changes in mGluR_5_ protein or mRNA were detected in cocaine-withdrawn rats left in home cage without extinction training (Ghasemzadeh et al. 2011; Ben-Shahar et al. 2009), these data speak against the pharmacological properties of cocaine or drug motivation (see Twining et al. 2009).

We also show that cocaine withdrawal resulted in a rise in GluN2A subunit expression (without significant changes in GluN1 or GluN2B protein levels) in the prefrontal cortex which seems to be attributable to the previous drug exposure. So far, similar increase was demonstrated after non-contingent cocaine administration in rats (Ary and Szumlinski 2007) and such overexpression of GluN2A subunit finding may suggest that the NMDA receptor kinetics of currents deactivation are faster than in controls (Yashiro and Philpot 2008). Such changes may also be associated with memory impairment (Jacobs and Tsien 2014) or enhanced cue and contextual fear conditioning (Gilmartin et al. 2013).

There was a significant (>50 %) cocaine-dependent increase in mGluR_5_ protein level in the rat hippocampus at 10-day withdrawal and this is the first report showing changes in protein levels in the rat hippocampus after extinction from cocaine iv delivery. In parallel to mGluR_5_, we observed a significant elevation of GluN1 subunit level in both cocaine groups, while GluN2A and GluN2B protein expression reached level of significance only in rats that previously self-administered cocaine. As recently shown, GluN1-2A-containing NMDA receptors mediate the formation of lasting contextual and trace fear memory and are more prominent to induce LTP in pyramidal neurons of the CA1 hippocampus (Jin and Feig 2010), while GluN1-2B-containing NMDA receptors are required for mediation trace fear conditioning (Gao et al. 2010). At the same time, 2-week cocaine forced abstinence inhibited proliferation in hippocampal cells (Yamaguchi et al. 2002; Garcia-Fuster et al. 2011) what may be directly linked with local reduction in Glu transmission (Sultan et al. 2013). In other words, changes in hippocampal mGluR_5_ and GluN1 proteins and in GluN2A and GluN2B subunits may reflect compensatory mechanisms of cocaine-mediated disturbed neurogenesis and memory processes in hippocampal cells.

Increased accumbal mGluR_5_ and GluN1 (but not GluN2A and GluN2B) receptor expression following extinction training was another finding in this study. Extinction from cocaine self-administration reorganized postsynaptic mGluR_5_ receptors in the nucleus accumbens subregions (not dissected in the present paper) with reduction in the accumbal shell (Ghasemzadeh et al. 2009c) but no changes in the accumbal core (Knackstedt et al. 2013) or raised (Ghasemzadeh et al. 2009b) after long access (6-hr daily) to cocaine self-administration. Neuroanatomical analyses with local microinfusions confirm the significance of mGluR_5_ receptor activity localized to accumbal core in controlling cocaine seeking (Backstrom and Hyytia 2007; Wang et al. 2013). A decline in accumbal (Swanson et al. 2001; Hao et al. 2010; Huang et al. 2011) mGluR_5_ protein expression was seen after withdrawal from non-contingent repeated administration of cocaine in rodents, similar to what others reported following a single passive administration of cocaine (Fourgeaud et al. 2004). Whether such increase reflects new learning processes due to previous unreinforced cocaine administration that occurred in those animals (see above) needs to be determined in additional studies.

The present paper found around a 200 % enhancement in the accumbal GluN1 protein expression after 10-day extinction training sessions and this increase was response-independent and is in line with past studies reporting non-contingent cocaine administration (Scheggi et al. 2002; Schumann and Yaka 2009). The nature of the relationship between GluN1 receptor state-dependent plasticity and response output (incubation?) during cocaine extinction is unresolved (see: Ghasemzadeh et al. 2009c). The lack of significant changes in striatal GluN2 and GluN2B subunit expression due to cocaine self-administration and extinction training extends recent findings in animals with short and long access to self-administered cocaine followed by 10-day home-cage withdrawal (Ben-Shahar et al. 2009).

As shown in this study, extinction training evoked a significant reduction in mGluR_5_ and GluN1 protein expression, with no changes in GluN2A and GluN2B subunits, in the dorsal striatum of rats with previous cocaine self-administration, but not in yoked cocaine animals. A drop in striatal mGluR_5_ expression during extinction learning was demonstrated through molecular (reduction in mGluR_5_ surface expression) and pharmacological (mGluR_5_ receptor ligands) analyses in rats that previously underwent cocaine self-administration (Knackstedt et al. 2013). At the same time, non-contingent repeated cocaine administration increased mGluR_5_ mRNA levels and/or protein expression (Ghasemzadeh et al. 1999; Lee et al. 2008) or did not alter NMDA receptor subunits (GluN1, GluN2A, and GluN2B) (Yamamoto and Zahniser 2012) in the rat dorsal striatum. It is not answered whether the observed molecular processes of the Glu signaling at mGluR_5_ and NMDA receptors in the dorsal striatum are directly linked to messed contextual encoding of extinction or enhanced extinction consolidation.

We also found altered expression (20 % decrease) of the scaffolding protein Homer1b/1c in the dorsal striatum of rats that underwent extinction training after cocaine self-administration. Of note, the same group of animals shows also a potent reduction in striatal mGluR_5_. Significant changes in striatal Homer1b/1c proteins during extinction from cocaine self-administration were observed previously under different experimental conditions (Ghasemzadeh et al. 2009c; Ben-Shahar et al. 2009) and such consistent changes may indicate extinction training as a trigger of Glu plasticity in the dorsal striatum, a structure critical for the learning and maintenance of goal-directed responding (Koob and Swerdlow 1988). Extinction training disrupts previously learned contextual encoding of extinction or enhances extinction consolidation (cf. Torregrossa et al. 2013); whether or not these processes offer a strategy to reduce cocaine relapse remains to be determined.

Withdrawal from cocaine induces decrease in basal synaptic Glu levels (e.g., Wydra et al. 2013) and in mechanisms responsible for Glu clearance that is different in various brain structures (see Baker et al. 2002; Melendez et al. 2005; Cavelier and Attwell 2005, Madayag et al. 2007, Pendyam et al. 2009; Knackstedt et al. 2010). It is still unknown whether differences in Glu levels and region-dependent mechanisms maintaining the neurotransmitter level are responsible for the changes in receptor expression observed in the present paper following cocaine self-administration. The total receptor amount, which is determined by a balance of protein synthesis (enhanced transcription and translation) and degradation (e.g., ubiquitination and proteasome-mediated degradation) was not addressed in this study as well as we also cannot answer if the labeled proteins were localized to the membrane surface.

## Summary

Our results showed that cocaine self-administration and its extinction training produce numerous alterations in mGluR_5_, NMDA, and Homer1b/1c protein expression that are region specific and are dependent of the manner (contingent or non-contingent) of cocaine administration. Extinction training procedure by withholding cocaine injections and cue-contingent presentations led to diminishment in the active lever pressing that paralleled an increase in the hippocampal GluN2A and B subunit and in the accumbal mGluR_5_. The latter procedures provoked also simultaneous decrease in mGluR_5_ and Homer1b/1c protein expression in the dorsal striatum but in other structures such correlation was not detected. These results do not also show correlation between changes in mGluR_5_ and NMDA protein expression via Homer1b/1c protein.

## Abbreviations

EMCDDA: European Monitoring Center for Drugs and Drug Addiction
UNDCP: United Nations Office on Drugs and Crime
NMDAR: N-methyl-D-aspartate receptors
Glu: Glutamate
LTP: Long-term potentiation
LTD: Long-term depression
FR: Fixed ratio
DA: Dopamine
NA: Norepinephrine
5-HT: Serotonin
